# Factors associated with chronic back pain in adults in Brazil

**DOI:** 10.1590/S1518-8787.2017051000052

**Published:** 2017-06-01

**Authors:** Deborah Carvalho Malta, Max Moura de Oliveira, Silvânia Suely Caribé de Araújo Andrade, Waleska Teixeira Caiaffa, Maria de Fatima Marinho de Souza, Regina Tomie Ivata Bernal

**Affiliations:** I Departamento de Enfermagem Materno Infantil e Saúde Pública. Escola de Enfermagem. Universidade Federal de Minas Gerais. Belo Horizonte, MG, Brasil; II Programa de Pós-Graduação. Faculdade de Saúde Pública. Universidade de São Paulo. São Paulo, SP, Brasil; III Diretoria de Vigilância de Doenças e Agravos Não Transmissíveis. Secretaria de Vigilância em Saúde. Ministério da Saúde. Brasília, DF, Brasil; IV Observatório de Saúde Urbana de Belo Horizonte. Departamento de Medicina Preventiva e Social. Faculdade de Medicina. Universidade Federal de Minas Gerais. Belo Horizonte, MG, Brasil; V Núcleo de Pesquisas Epidemiológicas em Nutrição e Saúde. Universidade de São Paulo. São Paulo, SP, Brasil

**Keywords:** Adult, Low Back Pain, epidemiology, Diagnostic Self Evaluation, Risk Factors, Socioeconomic Factors, Health Surveys, Adulto, Dor Lombar, epidemiologia, Autoavaliação Diagnóstica, Fatores de Risco, Fatores Socioeconômicos, Inquéritos Epidemiológicos

## Abstract

**OBJECTIVE:**

To identify associations of chronic back pain with sociodemographic characteristics, lifestyles, body mass index, self-reported chronic diseases and health assessment, according to sex.

**METHODS:**

We analyzed data from the 2013 National Health Survey, estimated the prevalence and their respective 95% confidence intervals (95%CI) of chronic back pain, according to selected variables and performed adjustment by age and education.

**RESULTS:**

18.5% of the Brazilian population reported chronic back pain, 15.5% (95%CI 14.7–16.4) of them being men and 21.1% (95%CI 20.2–22.0) being women. The characteristics that remained associated and statistically significant (p < 0.05) after adjustment, in men, were: age group, higher in men with 65 years or older (OR_a_ = 6.06); low education level; living in rural area; history of smoking, high salt intake, increase in the time of heavy physical activity at work and at home; being overweight (OR_a_ = 1.18) or obese (OR_a_ = 1.26); diagnostic of hypertension (OR_a_ = 1.42), high cholesterol (OR_a_ = 1.60); and worse health assessment in comparison with very good (good [OR_a_ = 1.48]; regular [OR_a_ = 3.22]; poor [OR_a_ = 5.00], very poor [OR_a_ = 8.60]). Among women, they were: increase with age, higher among women with 55-64 years (OR_a_ = 3.64); low education level; history of smoking, regular candy consumption, high salt intake, heavy physical activity at work and at home and increase in the time of these activities; being overweight (OR_a_ = 1.23) or obese (OR_a_ = 1.32); diagnosis of hypertension (OR_a_ = 1.50), high cholesterol (OR_a_ = 1.84); and worse health assessment than very good (good [OR_a_ = 1.43]; regular [OR_a_ = 3.16]; poor [OR_a_ = 5.44], very poor [OR_a_ = 8.19]).

**CONCLUSIONS:**

Our findings point out differences by sex and contribute to the knowledge of the panorama of chronic back pain, which, besides affecting individuals, generate negative socioeconomic impacts, by causing work-related disabilities and hindering everyday activities.

## RESUMO

**OBJETIVO:**

Identificar associações de dor crônica na coluna com características sociodemográficas, estilos de vida, índice de massa corporal, doenças crônicas autorreferidas e avaliação do estado de saúde, segundo sexo.

**MÉTODOS:**

Foram analisados dados da Pesquisa Nacional de Saúde 2013; estimadas as prevalências e seus respectivos intervalos de 95% de confiança (IC95%) da dor crônica na coluna, segundo variáveis selecionadas; e realizado ajuste por idade e escolaridade.

**RESULTADOS:**

18,5% da população brasileira referiram dor crônica na coluna, sendo 15,5% (IC95% 14,7–16,4) em homens e 21,1% (IC95% 20,2–22,0) em mulheres. As características que se mantiveram associadas após o ajuste e estatisticamente significativas (p < 0,05) em homens foram: aumento com a faixa etária, sendo maior entre aqueles com 65 anos ou mais (OR_a_ = 6,06); baixa escolaridade; morar em área rural; histórico de tabagismo, consumo elevado de sal, aumento do tempo de prática de atividade física pesada no trabalho e atividade pesada no domicílio; ter sobrepeso (OR_a =_ 1,18) ou obesidade (OR_a =_ 1,26); diagnóstico de hipertensão (OR_a_= 1,42), colesterol elevado (OR_a =_ 1,60); e pior avaliação do estado de saúde (bom [OR_a =_ 1,48]; regular [OR_a =_ 3,22]; ruim [OR_a =_ 5,00], muito ruim [OR_a =_ 8,60]). Entre mulheres: aumento com a faixa etária, sendo maior entre as mulheres com 55–64 anos (OR_a =_ 3,64); menor escolaridade; histórico de tabagismo, consumo de doces regularmente, consumo elevado de sal, atividade e aumento do tempo de prática de atividade física pesada no trabalho e atividade pesada no domicílio; ter sobrepeso (OR_a =_ 1,23) ou obesidade (OR_a =_ 1,32); diagnóstico de hipertensão (OR_a =_ 1,50), colesterol elevado (OR_a =_ 1,84); e piora da avaliação do estado de saúde (bom [OR_a =_ 1,43]; regular [OR_a =_ 3,16]; ruim [OR_a =_ 5,44], muito ruim [OR_a =_ 8,19]).

**CONCLUSÕES:**

Os achados apontam diferenças por sexo e contribuem no conhecimento do panorama da dor crônica na coluna, que além de afetar o indivíduo, geram impactos socioeconômicos negativos, por ocasionar incapacidades relacionadas ao trabalho e realização de atividades cotidianas.

## INTRODUCTION

Chronic back pain is one of the commonly reported complaints by the adult population, causing disability, reduced functionality, and absence from work^1^. This condition includes neck pain, chest pain, sciatica, and low back pain, which can be caused by different musculoskeletal diseases, intervertebral disc disorders, spondylosis or radiculopathy, the two latter being the most frequent ones.^a^

The cost of these diseases is high, both by the demand for health services, tests, medications, physical therapy, hospitalizations, and surgeries and by the expenses resulting from sick leaves and early retirements^2^. Social Security data show high rates of disability retirement related to back pain in Brazil (about 30/100,000 taxpayers in 2007), the highest among men and in older individuals^3^.

Analyses of the National Household Sample Survey (PNAD) in 2003 have already indicated back pain as the most reported among the surveyed chronic diseases, affecting 13.2% of the adult population^4^. In PNAD 2008, it was reported by 13.5% of adults, presenting itself as the second most reported cause, being more prevalent in women and overcoming the prevalence of 30% after 50 years of age and in individuals with low education level^5^.

It is estimated that from 70% to 85% of the population will have some episode of back pain in the course of life, which can be caused by anatomophysiological changes, as the wear on the musculoskeletal components of spinal support, pregnancy^2,6,7^, inflammatory, degenerative and neoplastic processes, birth defects (lordosis, kyphosis, muscle weakness), in addition to external causes (traffic accidents, falls, among others)^b^. The literature indicates a set of factors associated with chronic back pain, such as demographics factors (age, sex, income, and education), lifestyles (smoking and low physical activity, or strenuous physical work), and metabolic risk factors (obesity and other chronic diseases)^2,c^.

In 2013, chronic back problems were investigated on the National Health Survey (PNS), making it possible to analyze these data along with other health conditions and sociodemographic data that represent the Country. This study aimed to identify associations of chronic back pain with sociodemographic characteristics, lifestyles, body mass index, self-reported chronic diseases and health assessment, according to sex.

## METHODS

This is a cross-sectional study with data from the National Health Survey (PNS) of 2013, conducted by the Brazilian Institute of Geography and Statistics (IBGE) in partnership with the Brazilian Ministry of Health. PNS represents adults living in private households in urban and rural areas of the Country, in the five geographical macroregions, in the 27 Federative Units (UF), and in the Brazilian capitals^8,d^.

The sampling was conglomerated in three stages. In the first stage, we selected the census tracts; in the second stage, the households; and in the third stage, a resident with 18 years or older. The final sample was composed by 64,348 households, with 60,202 conducted interviews. We performed a weighting considering weights for each stage of selection of the sample and for non-response, so that the sample could represent the Country and the studied geographic strata^8,d^.

The data were collected using handheld computers (Personal Digital Assistance). The module on chronic diseases was answered by the adult selected for individual interview^8,d^.

The outcome examined in this study was the prevalence of chronic back pain, measured by the question: “Do you have a chronic back problem, such as chronic back or neck pain, low back pain, sciatica, vertebrae or disc problems?” The response options were “yes” or “no.”

The explanatory variables were: a) sociodemographic characteristics: sex; age group in years (18–24, 25–34, 35–44, 45–54, 55–64, 65 or older); education level; race or skin color (white, black, brown); area of residence (urban, rural); b) lifestyles as risk and protective factors: smoking; abusive consumption of alcohol^e^; consumption of red meat with fat; regular candy consumption; regular soda consumption^f^; high salt intake; recommended consumption of fruits and vegetables – five or more daily servings^g^; insufficient physical activity in four areas – recreation, work, displacement, and domestic activities; heavy physical activity at work – time (minutes) of heavy physical activity at work in a normal day (0, 1-149, 150 or more); heavy physical activity at home – time (minutes) of heavy physical activity at home in a normal day (0, 1-149, 150 or more); watching TV for more than three hours; c) metabolic risk factors: body mass index category (eutrophy, overweight, obesity); self-reported chronic disease: hypertension, diabetes, and high cholesterol; d) health assessment (very good, good, regular, poor, and very poor).

We estimated the prevalence of back pain and respective 95% confidence intervals (95%CI) according to the studied explanatory variables. We estimated the odds ratio (OR) and respective 95%CI crude and adjusted for age and education. The adjustment was carried out to control bias in the estimates, since the literature points out significant differences regarding these variables^5^. The analysis was conducted in the survey module for complex samples of the Stata program, version 12.1 (StataCorp., CollegeStation, EUA).

The PNS project was approved by the Research Ethics Committee of the Conselho Nacional de Saúde (Process 328,159, June 26, 2013). All participants signed the informed consent form.

## RESULTS

In Brazil, chronic back pain was reported by 18.5% of adults: 15.5% (95%CI 14.7–16.4) in men and 21.1% (95%CI 20.2–22.0) in women. In analyzing the chronic back pain, we observed that its prevalence differed according to sex. In both sexes, we verified differences in the reported prevalence with increasing age, low education level, smoking history, heavy activity at work and at home (and the increase in time of these activities), overweight and obesity, worsening of back pain when the health assessment gets worse, and history of hypertension, diabetes, or high cholesterol (Table 1).

**Table 1:** Prevalence (%) and respective confidence interval (95%CI) of people who reported chronic back pain, according to selected variables, by sex. National Health Survey, Brazil, 2013.

Variable	Male			Female		
	
	%^a^	95%CI	p^b^	%^a^	95%CI	p^b^
Age group (years)			< 0.01			< 0.01
18–24	5.6	4.4–6.8		10.1	8.3–12	
25–34	9.9	8.6–11.2		12.7	11.4–14	
35–44	14.7	13–16.3		20	18.3–21.7	
45–54	21.6	19.5–23.8		26.8	24.8–28.9	
55–64	22.3	19.8–24.8		30.4	27.8–32.9	
65 or older	25.5	22.6–28.4		31.3	28.9–33.7	
Education level			< 0.01			< 0.01
Illiterate/Some elementary or middle school	21.9	20.4–23.4		27.1	25.6–28.5	
Elementary or middle school/Some high school	12.5	10.7–14.3		19.1	17.1–21.1	
High school/Some higher education	10.5	9.3–11.8		16.9	15.6–18.1	
Higher education degree	12	9.8–14.2		16.7	14.7–18.8	
Race/skin color^c^			0.6			0.02
White	15.9	14.6–17.1		22.2	20.9–23.5	
Black	14.4	11.8–17		20.9	18.3–23.5	
Brown	15.5	14.4–16.7		19.8	18.5–21	
Area			< 0.01			0.43
Urban	14.6	13.7–15.5		20.9	20–21.9	
Rural	20.7	18.7–22.8		21.9	19.6–24.3	
Smoking			< 0.01			< 0.01
Non-smoker	11.8	10.8–12.8		18.5	17.6–19.5	
Former smoker	23	21–24.9		30.2	27.8–32.6	
Smoker	19	17.1–20.9		26.6	24.1–29.2	
Abusive consumption of alcohol			0.47			0.16
No	15.6	14.8–16.5		21.2	20.2–22.1	
Yes	14.7	12.5–17		17.8	13.4–22.2	
Consumption of red meat with fat			0.05			0.21
Yes	16.4	15.2–17.7		20.3	18.7–21.8	
No	14.9	13.9–15.9		21.4	20.3–22.4	
Regular candy consumption			0.73			0.18
No	15.5	14.6–16.4		21.4	20.4–22.4	
Yes	15.8	14–17.7		20.1	18.4–21.7	
Regular soda consumption			< 0.01			0.01
No	16.3	15.3–17.3		21.6	20.6–22.6	
Yes	13.4	11.8–15		18.9	17.2–20.7	
High salt intake			0.36			0.62
No	15.4	14.5–16.3		21.1	20.2–22.1	
Yes	16.3	14.4–18.3		20.6	18.5–22.7	
Recommended consumption of fruits and vegetables (five or more daily servings)			0.38			< 0.01
Yes	16.5	14.2–18.7		24.1	22–26.1	
No	15.4	14.5–16.3		20.4	19.5–21.4	
Insufficiently physical active in the four areas			< 0.01			0.18
No	15.4	14.4–16.4		21.6	20.4–22.9	
Yes	15.8	14.4–17.1		20.6	19.4–21.7	
Heavy activity at work			< 0.01			< 0.01
No	11.7	10.7–12.7		17.6	16.3–18.8	
Yes	18.7	17–20.5		27.7	24.9–30.6	
Time (minutes) of heavy physical activity at work in a normal day			< 0.01			< 0.01
0	14.9	14–15.7		19.8	18.8–20.7	
1–149	17.9	14.1–21.8		23.5	20–27.1	
≥ 150	25	20.9–29		25.9	23.9–27.9	
Heavy physical activity at home			< 0.01			< 0.01
No	14.9	14–15.7		19.8	18.8–20.7	
Yes	22	19.2–24.8		25.4	23.5–27.2	
Time (minutes) of heavy physical activity at home in a normal day			< 0.01			< 0.01
0	14.5	13.5–15.4		20.4	19.5–21.4	
1–149	16	11.8–20.2		27.8	21.5–34.1	
≥ 150	19.1	17.3–21		27.7	24.5–30.9	
Watching TV for more than three hours			0.15			0.76
No	15.9	14.9–16.9		21.2	20.1–22.2	
Yes	14.6	13.1–16.1		20.9	19.5–22.3	
Classification by BMI^d^			0.09			< 0.01
Normal	15.4	14–16.8		18	16.6–19.3	
Overweight	16.3	14.9–17.8		23.5	21.6–25.4	
Obesity	18.3	15.9–20.8		25.6	23.3–28	
Hypertension			< 0.01			< 0.01
No	13.5	12.7–14.4		17.5	16.6–18.4	
Yes	24.6	22.3–26.8		32.3	30.4–34.2	
Diabetes			< 0.01			< 0.01
No	15.2	14.4–16.1		20.3	19.4–21.3	
Yes	21.1	17.3–24.9		30.8	27.5–34	
Cholesterol			< 0.01			< 0.01
No	14.5	13.6–15.3		18.4	17.5–19.3	
Yes	25.7	22.7–28.7		36.1	33.7–38.5	
Health assessment			0.68			< 0.01
Very good	6.8	5.4–8.3		10.2	8.4–11.9	
Good	11.2	10.3–12.2		14.6	13.6–15.6	
Regular	25.6	23.7–27.5		30.4	28.7–32.1	
Poor	37.5	32.7–42.2		45	41.1–48.9	
Very poor	49.6	39.8–59.4		52.5	45.1–59.9	
Total	15.5	14.7–16.4		21.1	20.2–22	

BMI: body mass index.

^a^ Weighted estimates: selected resident’s weight with correction of non-interview with calibration by population projection for the selected resident.

^b^ Chi-square Test.

^c^ Excluding the categories yellow and indigenous.

^d^ 32% missing data for BMI.

Only in men, we observed higher prevalence of chronic back pain among those who live in rural areas, who consume red meat, and who are insufficiently active in the four areas. In women, we found higher prevalence among those who self-reported as white and those with recommended consumption of fruits and vegetables (Table 1).

Tables 2 and 3 present the estimates of the crude and adjusted OR by age and education, according to sex. After the adjustment, the characteristics that remained associated and statistically significant (p < 0.05) with back pain in men were: a) sociodemographic characteristics (all age groups over 25 years, being higher at 65 years or older; low education level; living in the rural area); b) lifestyles (being a smoker or a former smoker; high salt intake; recommended consumption of fruits and vegetables; heavy activity at work and at home, and the increase in time of these activities); c) metabolic risk factors (being overweight or obese; diagnosis of hypertension; high cholesterol); d) worse health assessment when compared with very good assessment (good, regular, bad, very bad). Being insufficiently physically active protected men from chronic back pain (Table 2).

**Table 2:** Associated factorsa (crude and adjusted OR and respective 95%CI) in men who reported chronic back pain, according to selected variables. National Health Survey, Brazil, 2013.

Variable	OR_crude_	95%CI	OR_a_^b^	95%CI
Age group (years)				
18–24	1		1	
25–34	1.84	1.41–2.42	1.87	1.42–2.46
35–44	2.9	2.25–3.73	2.73	2.11–3.53
45–54	4.65	3.59–6.02	4.2	3.22–5.47
55–64	4.83	3.68–6.33	4.16	3.16–5.49
65 or older	5.78	4.39–7.62	4.57	3.45–6.06
Education level				
Illiterate/Some elementary or middle school	1		1	
Elementary or middle school/Some high school	0.51	0.42–0.61	0.7	0.58–0.85
High school/Some higher education	0.42	0.36–0.49	0.59	0.51–0.7
Higher education degree	0.49	0.39–0.61	0.54	0.43–0.67
Race/skin color^c^				
White	1		1	
Black	0.9	0.71–1.13	0.84	0.67–1.05
Brown	0.98	0.87–1.11	0.98	0.86–1.12
Area				
Urban	1		1	
Rural	1.53	1.32–1.76	1.26	1.08–1.46
Smoking				
Non-smoker	1		1	
Former smoker	2.23	1.94–2.56	1.54	1.34–1.78
Smoker	1.75	1.5–2.05	1.41	1.2–1.65
Abusive consumption of alcohol				
No	1		1	
Yes	0.93	0.77–1.13	1.06	0.87–1.28
Consumption of red meat with fat				
Yes	1		1	
No	0.89	0.79–1	0.92	0.82–1.03
Regular candy consumption				
No	1		1	
Yes	1.03	0.88–1.2	1.18	1–1.38
Regular soda consumption				
No	1		1	
Yes	0.79	0.68–0.93	1.01	0.86–1.18
High salt intake				
No	1		1	
Yes	1.07	0.92–1.25	1.28	1.09–1.49
Recommended consumption of fruits and vegetables (five or more daily servings)				
Yes	1		1	
No	0.92	0.78–1.1	0.99	0.83–1.19
Insufficiently physical active in the four areas				
No	1		1	
Yes	1.03	0.91–1.16	0.83	0.72–0.94
Heavy activity at work				
No	1		1	
Yes	1.73	1.5–2	1.65	1.41–1.92
Time (minutes) of heavy physical activity at work in a normal day				
0	1		1	
1–149	1.25	0.96–1.62	1.33	0.96–1.84
≥ 150	1.9	1.52–2.38	1.52	1.31–1.77
Heavy physical activity at home				
No				
Yes	1.61	1.36–1.92	1.6	1.33–1.92
Time (minutes) of heavy physical activity at home in a normal day				
0	1		1	
1–149	1.13	0.82–1.55	1.3	0.99–1.72
≥ 150	1.4	1.22–1.61	1.81	1.44–2.29
Watching TV for more than three hours				
No	1		1	
Yes	0.9	0.79–1.04	0.95	0.82–1.09
BMI^d^				
Normal	1		1	
Overweight	1.15	1.01–1.31	1.18	1.02–1.36
Obesity	1.32	1.11–1.58	1.26	1.05–1.53
Hypertension				
No	1		1	
Yes	2.08	1.82–2.39	1.42	1.23–1.65
Diabetes				
No	1		1	
Yes	1.49	1.18–1.88	0.98	0.77–1.23
Cholesterol				
No	1		1	
Yes	2.04	1.72–2.42	1.6	1.34–1.92
Health assessment				
Very good	1		1	
Good	1.73	1.36–2.19	1.48	1.16–1.89
Regular	4.68	3.64–6.02	3.22	2.49–4.16
Poor	8.16	6.04–11.01	5	3.67–6.81
Very poor	13.38	8.52–21.02	8.6	5.51–13.44

BMI: body mass index.

^a^ Weighted estimates: selected resident’s weight with correction of non-interview with calibration by population projection for the selected resident.

^b^ OR adjusted by age and education.

^c^ Excluding the categories yellow and indigenous.

^d^ 32% missing data for BMI.

**Table 3:** Associated factorsa (crude and adjusted OR and respective 95%CI) in women who reported chronic back pain, according to selected variables. National Health Survey, Brazil, 2013.

Variable	OR_crude_	95%CI	OR_a_^b^	95%CI
Age group (years)				
18–24	1		1	
25–34	1.29	1.03–1.62	1.31	1.04–1.65
35–44	2.22	1.76–2.79	2.2	1.74–2.78
45–54	3.25	2.61–4.06	3.11	2.48–3.91
55–64	3.86	3.07–4.87	3.64	2.86–4.62
65 or older	4.04	3.23–5.05	3.62	2.86–4.59
Education level				
Illiterate/Some elementary or middle school	1		1	
Elementary or middle school/Some high school	0.64	0.55–0.74	0.9	0.77–1.04
High school/Some higher education	0.55	0.49–0.61	0.82	0.73–0.93
Higher education degree	0.54	0.46–0.64	0.68	0.58–0.8
Race/skin color^c^				
White	1		1	
Black	0.93	0.78–1.1	0.88	0.74–1.06
Brown	0.86	0.78–0.95	0.88	0.79–0.97
Area				
Urban	1		1	
Rural	1.06	0.92–1.23	1	0.85–1.17
Smoking				
Non-smoker	1		1	
Former smoker	1.9	1.68–2.15	1.46	1.28–1.65
Smoker	1.59	1.38–1.84	1.32	1.13–1.53
Abusive consumption of alcohol				
No	1		1	
Yes	0.81	0.6–1.09	1.04	0.76–1.43
Consumption of red meat with fat				
Yes	1		1	
No	1.07	0.96–1.19	1.05	0.94–1.17
Regular candy consumption				
No	1		1	
Yes	0.92	0.82–1.04	1.14	1.01–1.29
Regular soda consumption				
No	1		1	
Yes	0.84	0.75–0.96	1.05	0.92–1.19
High salt intake				
No	1		1	
Yes	0.97	0.84–1.11	1.18	1.02–1.36
Recommended consumption of fruits and vegetables (five or more daily servings)				
Yes	1		1	
No	0.81	0.72–0.91	0.85	0.75–0.96
Insufficiently physical active in the four areas				
No	1		1	
Yes	0.94	0.86–1.03	0.84	0.76–0.92
Heavy activity at work				
No	1		1	
Yes	1.8	1.54–2.11	1.69	1.44–2
Time (minutes) of heavy physical activity at work in a normal day				
0	1		1	
1–149	1.25	1.02–1.53	1.71	1.23–2.37
≥ 150	1.42	1.27–1.59	1.64	1.38–1.94
Heavy physical activity at home				
No	1		1	
Yes	1.38	1.24–1.54	1.48	1.32–1.66
Time (minutes) of heavy physical activity at home in a normal day				
0	1		1	
1–149	1.5	1.09–2.06	1.31	1.05–1.63
≥ 150	1.49	1.26–1.76	1.54	1.36–1.73
Watching TV for more than three hours				
No	1		1	
Yes	0.98	0.89–1.09	0.96	0.87–1.07
BMI^d^				
Normal	1		1	
Overweight	1.28	1.13–1.44	1.23	1.09–1.39
Obesity	1.43	1.26–1.63	1.32	1.15–1.51
Hypertension				
No	1		1	
Yes	2.25	2.04–2.49	1.5	1.34–1.68
Diabetes				
No	1		1	
Yes	1.74	1.49–2.03	1.11	0.94–1.31
Cholesterol				
No	1		1	
Yes	2.5	2.23–2.81	1.84	1.63–2.06
Health assessment				
Very good	1		1	
Good	1.51	1.23–1.85	1.43	1.16–1.76
Regular	3.86	3.14–4.74	3.16	2.55–3.91
Poor	7.24	5.65–9.29	5.44	4.21–7.03
Very poor	9.78	6.89–13.87	7.19	4.95–10.44

BMI: body mass index.

^a^ Weighted estimates: selected resident’s weight with correction of non-interview with calibration by population projection for the selected resident.

^b^ OR adjusted by age and education.

^c^ Excluding the categories yellow and indigenous.

^d^ 32% missing data for BMI.

After the adjustment, the variables that remained associated and statistically significant (p < 0.05) with back pain in women were: a) sociodemographic characteristics (all age groups over 25 years, being higher after 55 years old; low education level); b) lifestyles (being smoker or former smoker; high candy consumption; high salt intake; heavy activity at work and at home, and the increase in time of these activities); c) metabolic risk factors (being overweight or obese; diagnosis of hypertension; high cholesterol); d) worse health assessment when compared with very good assessment (good, regular, poor, very poor). Self-declaring as brown, being insufficiently physically active in the four areas, and having the recommended consumption of fruits and vegetables protected women from back pain (Table 3).

In both sexes, the variables abusive consumption of alcohol, red meat or soda, watching TV for more than three hours and diagnosis of diabetes were not associated with the outcome (Tables 2 and 3).

## DISCUSSION

PNS data show that, approximately, one-fifth of the Brazilian population reported chronic back pain. The characteristics associated with higher prevalence of chronic back pain, after the proposed adjustment, in both sexes, were: increasing age; low education level; smoking history; high salt intake; heavy activity at work or at home, and the increase in the time spent on these activities; being overweight or obese; having chronic diseases such as hypertension and high cholesterol; and worse health assessment than very good (good, regular, poor, very poor). In men, living in the rural area remained associated with chronic back pain. In women, the variables brown race/skin color, regular candy consumption, and recommended consumption of fruits and vegetables remained associated as protective factors. In addition, being insufficiently active in the four areas of physical activity protected both sexes from back pain.

The high prevalence of chronic back pain results in limitation of activities, high demand for health services^2,9^, and, consequently, high social costs, such as reduction of productivity, absenteeism at work and social security expenses^7,9,10^. PNS showed a higher prevalence of chronic back pain than those estimated by PNAD 2003 and 2008^4,5^.

Women were those who most reported chronic back pain, which has been attributed to the greater awareness of women about the symptoms and signs of diseases^2,5^. Factors such as performing housework in greater intensity, which remained associated after the adjustment, increased exposure to repetitive work, non-ergonomic position, and work at high speed are also mentioned in the literature^2,7^. In addition, the differences in the anatomical and functional characteristics of women, such as smaller height, less muscle mass, less bone mass, joints more fragile and less adapted to strenuous physical effort, may result in more overload in the back^2,11^. Studies also mention pregnancy and postpartum as factors that explain the higher prevalence of back pain among women. In pregnancy, hormones such as relaxin, estrogen, and progesterone, increase the flexibility of the ligaments of the spine and hip, which could lead to lordosis, muscle contractures (because of the weight increase), and posture (because of the fetal growth). In the postpartum, back pain can be related to postural inadequacies while breastfeeding, to the child’s weight and other factors^2,12^.

Age was an important predictor in crude and adjusted analyses, in both sexes, indicating that the prevalence of chronic pain increases gradually and proportionally with increasing age. This relationship has also been observed in studies with data from PNAD 2003 and 2008^4,5^. The observed dose-response can be explained by changes in the body due to the aging process, such as postural problems, reduced flexibility, greater musculoskeletal degeneration, and, consequently, worsening pain. In addition, the back pain found in middle-aged adults (40 to 49 years), an economically active age group, may be associated with work activities^2,13,14,15,a^.

Low education level and income have also been considered predictive of the development of chronic pain^14,15^. PNAD also observed that less educated individuals had more chronic pain^5^. Studies conducted in the South region of the Country^7^ and in Bambuí (Minas Gerais)^12^ found association between back pain and low education level. This can be a result of professions that require strenuous work, more physical activity, and less health-care, which are common in populations with less schooling. In this study, similarly, low education level was associated with back pain in both sexes. Still, men living in rural areas had higher association with back pain, which can be explained by the more strenuous work in rural areas.

This study found that brown women were protected against back pain. Although Webb et al.^15^ found increased risk in Asians, race and ethnicity have not been reported as factors associated with the presence of chronic pain^13^. The self-reported race/skin color may have influenced the results found here. Therefore, these data need to be confirmed in further studies.

Evidence show that smokers and former smokers are more prone to develop chronic pain^13,15,16^, because nicotine could lead to an activation of the immune system, predisposing to rheumatic diseases and back pain, among other conditions. This study found higher prevalence of back pain in smokers and former smokers, in both sexes, both in the crude analysis and after adjustment for age and education.

The outcome remained associated with intense or heavy physical activity at work or at home, in both sexes. Heavy physical activity is not considered beneficial to health, because it is associated with fatigue, muscle and joint overload, leading to musculoskeletal problems^2^.

The chronic back problem limits usual activities, which is worrisome, since these limitations affect adults in the economically active age group and decrease functional capacity at work and in daily activities, interfering in their quality of life^17^.

This study also indicated, for both sexes, that overweight and obesity are associated with chronic back pain, confirming other studies^13,16-18^. Increased weight leads to muscle overload, as well as to inflammatory processes in the bones and spinal disc damage, favoring the onset of low back pain and herniated disc, among other back diseases. Public policies aiming the reduction of obesity should be prioritized^13,18^, because weight loss can decrease pain and disability.

The presence of chronic diseases, such as hypertension and high cholesterol, was also associated with chronic back pain in this study. This may be related to the aging process, since there is greater risk of comorbidities with increasing age^4,5^. We also observed that, the worse the health self-assessment, the greater the association with chronic back problem. Studies show that a worse health self-assessment is associated with worse health outcomes (including chronic back pain) and higher morbidity, and these characteristics were present in both sexes^2,19^.

The adequate consumption of fruits and vegetables helps in maintaining appropriate concentration levels of micronutrients, such as vitamin K. Low consumption increases the risk of fractures and less bone mass^20^; however, in this study, we found an inverse association in women. A possible explanation is that the increased consumption of fruits and vegetables has occurred after the back pain, as orientation to healthy nutrition and weight control, resulting in reverse causality.

Cross-sectional studies as this are advantageous regarding quickness and low cost. However, there are inherent limitations to this design, such as the possibility of reverse causality. In this study, this characteristic may have affected the associations found between the studied outcome and the variables related to lifestyle and medical diagnoses; however, we believe not in the case of associations related to socioeconomic variables.

In addition, there is the possibility of overestimation of prevalence, since the chronic pain was self-reported. The recognition of the disease by the individual depends on the degree of perception, frequency of signs and symptoms, and access to medical services, health professionals, and diagnostic tests.

Associations tended to be similar between sexes, indicating that back problems are related to the studied factors, with great magnitude and regardless of sex for some, except for the variables race/skin color and area of residence. Thus, our findings contribute to the knowledge of the panorama of these diseases that, besides affecting the individual, generate negative socioeconomic impacts, especially by causing work-related disabilities and hindering everyday activities. Specific health interventions are needed for the population groups that have higher prevalence of back problem.

## Footnotes

a Ministério da Previdência Social (BR). Relação das 10 maiores frequências de auxílios-doença concedidos segundo os códigos da CID-10 – Acumulado Ano 2007.

b World Health Organization. Identification and control of work-related diseases: report of a WHO expert committee meeting, 1983, Geneva. Geneva; 1985. (WHO Technical Report Series, 714).

c National Institute for Occupational Safety and Health. Musculoskeletal disorders and workplace factors. Cincinnati: NIOSH; 1997. Available from: https://www.cdc.gov/niosh/docs/97-141/pdfs/97-141.pdf

d Instituto Brasileiro de Geografia e Estatística. Pesquisa Nacional de Saúde 2013: percepção do estado de saúde, estilos de vida e doenças crônicas – Brasil, Grandes Regiões e Unidades da Federação. Rio de Janeiro: IBGE; 2014 [cited 2015 Oct 8]. Available from: http://ftp.ibge.gov.br/PNS/2013/pns2013.pdf

e Intake of four or more portions, by women, or five or more portions, by men, on the same occasion, within the last 30 days.

f Consumption of soft drinks or artificial juices five or more days per week.

g Daily intake of at least 400 grams of fruits and vegetables, recommended by the World Health Organization, which amounts to about five daily portions of these foods.
